# Continental United States climate projections based on thermodynamic modification of historical weather

**DOI:** 10.1038/s41597-023-02485-5

**Published:** 2023-09-28

**Authors:** Andrew D. Jones, Deeksha Rastogi, Pouya Vahmani, Alyssa M. Stansfield, Kevin A. Reed, Travis Thurber, Paul A. Ullrich, Jennie S. Rice

**Affiliations:** 1 Climate and Ecosystem Sciences Division, Lawrence Berkeley National Laboratory, Berkeley, USA; 2 Energy and Resources Group, University of CA, Berkeley, USA; 3 Computational Sciences and Engineering Division, Oak Ridge National Laboratory, Oak Ridge, USA; 4 School of Marine and Atmospheric Sciences, Stony Brook University, Stony Brook, USA; 5 Department of Atmospheric Science, Colorado State University, Fort Collins, USA; 6 Earth Systems Science Division, Pacific Northwest National Laboratory, Richland, USA; 7 Department of Land, Air, and Water Resources, University of CA, Davis, USA; 8 Atmospheric Sciences and Global Change Division, Pacific Northwest National Laboratory, Richland, USA

**Keywords:** Projection and prediction, Climate and Earth system modelling

## Abstract

Regional climate models can be used to examine how past weather events might unfold under different climate conditions by simulating analogue versions of those events with modified thermodynamic conditions (i.e., warming signals). Here, we apply this approach by dynamically downscaling a 40-year sequence of past weather from 1980–2019 driven by atmospheric re-analysis, and then repeating this 40-year sequence a total of 8 times using a range of time-evolving thermodynamic warming signals that follow 4 80-year future warming trajectories from 2020–2099. Warming signals follow two emission scenarios (SSP585 and SSP245) and are derived from two groups of global climate models based on whether they exhibit relatively high or low climate sensitivity. The resulting dataset, which contains 25 hourly and over 200 3-hourly variables at 12 km spatial resolution, can be used to examine a plausible range of future climate conditions in direct reference to previously observed weather and enables a systematic exploration of the ways in which thermodynamic change influences the characteristics of historical extreme events.

## Background & Summary

Future climate projections are widely used across research and practitioner communities to provide insight into how climate change is affecting and will continue to affect environmental conditions of relevance to society, including the characteristics of extreme events such as heat waves, droughts, and storms. The recently completed Sixth Coupled Model Intercomparison Project (CMIP6)^1^ offers a rich dataset of coordinated global climate projections that spans multiple Global Climate Models (GCMs) and multiple time-evolving future scenarios^2^ framed around both socioeconomic conditions, known as Shared Socioeconomic Pathways (SSPs)^3^, and various levels of plausible anthropogenic forcing of the climate, known as Representative Concentration Pathways (RCPs)^4^.

Applying these projections for detailed analysis of climate impacts and adaptation, however, often requires that one contend with three interrelated challenges: downscaling, bias-correction, and selecting future projections that reflect an appropriate range of uncertainty. First is the challenge of downscaling relatively coarse global data to smaller spatial scales that are fit-for-purpose for local and regional scale analysis. While GCMs can be run at very high spatial resolutions^5–7^, the majority of projections available via CMIP6 are typically on the order of 100 km or greater in horizontal grid spacing. This resolution does not resolve regional processes affecting finer-scale heterogeneous conditions, particularly in regions of complex topography^8^ or other regions with variable surface features such as metropolitan areas^9^. Such regions can exhibit variability at scales of 10 km or less, depending on the phenomenon of interest. There are various downscaling methods, many of which also try to address the fact that GCMs are not perfect representations of reality and therefore may exhibit biases when evaluated against historical data^10^, although downscaling approaches can themselves introduce biases^11^. Future projections reflect a range of uncertainties arising from multiple emission scenarios, models, and natural variability^12,13^. However, with limited resources, it is often not possible to downscale or further analyse all available projections, requiring instead a careful consideration of how to sample the range of possibilities in a parsimonious yet representative manner^14^. Solutions to these intersecting challenges have distinct advantages and disadvantages, which make them suitable or unsuitable for different applications^15^.

One commonly employed technique for downscaling climate data to practitioner-relevant scales is statistical downscaling. This technique involves the identification of statistical relationships among larger scale variables represented by GCMs and smaller scale observed outcomes of interest within a historical period, which can then be extrapolated to estimate such smaller scale outcomes associated with future GCM projections^16–20^. Statistical downscaling has the advantage of being relatively computationally inexpensive and thus can be applied to a large number of projections. This enables a broad sampling across many models, model realisations, and future scenarios, which can then be sub-sampled post-hoc according to the needs of different applications. Statistical downscaling procedures also typically have a built-in bias-correction step^16,19^. However, statistical downscaling is limited to cases in which adequate observational data are available for establishing robust statistical relationships, which limits the number of variables that can be downscaled and the temporal resolution of the downscaled data (often daily rather than hourly). Statistical downscaling also implicitly assumes that the relationship among large- and small-scale variables is stationary, even in climate futures that are quite different from present day. This so-called “stationarity” assumption^21^ has been shown to break down in certain cases where substantial environmental change occurs, such as in mountainous regions where warming fundamentally alters snow regimes and therefore land-atmosphere coupling processes^22^. Moreover, if multiple variables are each statistically downscaled and bias-corrected independently of one another, then questions arise regarding how physically consistent the resulting suite of variables is with one another, although cases have been documented in which correlations among independently downscaled and bias-corrected variables are indeed preserved^17^, and multivariate bias-correction techniques have also been developed^23^.

A second technique is direct dynamical downscaling, which involves the application of regional climate models (RCMs) that take coarse-scale GCM data as boundary conditions to further simulate smaller scale physical processes^24–26^. Dynamical downscaling yields a large suite of variables (comparable to GCMs themselves), which can be saved at high temporal resolution, and which are guaranteed to be physically consistent with one another by the physical constraints built into RCMs. However, regional climate models introduce their own inherent biases and also do not explicitly correct the biases of the GCMs that are used for their inputs^15,27^. This means that projections derived from different GCM-RCM pairings generally simulate statistically distinct historical climates^26,28^, complicating their comparison with one another and often necessitating an additional bias-correction step to obtain a consistent baseline historical period and to render them more useful for impacts and adaptation applications^11,29^. Bias-correction of dynamically modelled hydrologic phenomena has been shown to correct certain statistical properties while exacerbating errors in others^30^. Additionally, the high computational cost and data requirements of dynamical downscaling mean that few overall projections can be downscaled as a practical matter, requiring careful a priori consideration of how to sub-sample within the larger ensemble of projections available for downscaling^14^.

More recently, Thermodynamic Global Warming (TGW) simulations have been developed as another method for leveraging the information within GCMs to gain insight about future climate conditions^31–37^. This approach attempts to first reproduce past sequences of weather events, and then applies a thermodynamic warming signal (e.g., changes in temperature and moisture) derived from one or more GCMs to examine how those same events would play out in a hotter climate. We note that variations on this approach have been referred to by several names in the literature, including “surrogate warming”^38^, “pseudo global warming”^35^, and “imposed global warming”^37^, although the specific application of the concept has differed. In our research, we adopt the moniker TGW as we believe it best describes the essential distinguishing feature of the approach as we’ve implemented it.

TGW commonly relies on RCMs to translate larger scale conditions to smaller scales^33–36^, and thus can be thought of as a specialised form of dynamical downscaling in such cases. However, rather than directly taking GCM outputs as boundary conditions, TGW simulations first begin by downscaling re-analysis data. Re-analysis is a method of assimilating weather observations into atmospheric models in order to produce an observationally consistent reproduction of past weather events^39,40^. Downscaling re-analysis thus yields a high-resolution baseline simulation that reproduces a known sequence of historical weather. This is notably distinct from directly downscaling historical GCM simulations from CMIP6^1^, which by design are not intended to reproduce individual past events. Next, the re-analysis data are modified by applying a thermodynamic climate change signal derived from GCMs (e.g., by increasing mean temperatures and absolute humidity in a manner consistent with the mean changes in one or more GCMs). This modified re-analysis data is then downscaled in the same way as the original data, yielding a simulation that can be interpreted as replaying past events in the context of future thermodynamic warming. The downscaling step is important for imposing physical consistency via the RCM, while also resolving finer scale processes. In this way, the TGW approach differs from simpler “delta” methods^41,42^, in which warming signals are added to historical data without any additional modelling.

TGW has been applied to examine discrete events, such as heat waves^37^, atmospheric rivers^32^, tropical cyclones^33^, and droughts^31,34^, to enable a careful examination of how these known, and often impactful, events might have unfolded if the same large-scale dynamical conditions occurred in the context of a warmer future climate. The approach has also been used in a climate change attribution framework to quantify the degree to which the characteristics of observed events have already been influenced by climate change^33,43^. Focusing on known events and their plausible analogues in alternative climates can be a useful approach that supports climate risk analysis and adaptation planning, due to the compatibility of this approach with decision-making processes aimed at learning from or preparing for particular events^44^, and may also support the quantification of losses and damages associated with anthropogenic influences on specific impactful events^45^. Furthermore, the TGW approach partitions uncertainty in a useful manner by focusing detailed mechanistic modelling on the better understood thermodynamic dimensions of climate change^46,47^, while allowing the less well understood dynamical aspects to be explored through a plausible physical climate scenario framework^44^. It is important to note that dynamical changes (e.g., changes in atmospheric circulation or large-scale modes of variability) influence the frequency with which weather patterns and events occur, and so the TGW approach is more suited to understanding how the characteristics and intensity of known events could change, rather than providing a complete picture of how their frequencies change.

Examining climate change implications on discrete events offers a unique perspective for understanding the processes driving changing event characteristics, but also raises questions about generalizability to a larger class of related events. A few studies have applied the TGW technique over sufficiently large temporal and spatial domains to examine a large number of extreme events of the same type. These include a study that applied the technique to 13 years of historical data covering the entire Continental United States (CONUS) land area^35^, and one that applied the technique to 15 summer months covering the state of California^36^. Such datasets have provided a valuable scientific resource, resulting in a number of studies examining the generalised implications of thermodynamic warming on different event types such as heat waves^36,48^, tropical cyclones^49^, and convective dynamics^50^.

In the present study, we aim to produce a set of CMIP6-derived climate projections for the continental US that can be applied in a consistent manner from past to future and across the range of sectors, spatial scales, and temporal scales required to assess climate risks and explore tradeoffs and interactions among adaptive responses^51^. In particular, we seek to produce a dataset that can be used to explore adaptive responses that evolve over the decadal timescales inherent to infrastructure planning and land use change, while also providing insight into shorter timescale extreme events that lead to acute stress on human systems. In doing so, we seek to leverage some of the useful features of the TGW approach such as reproduction of known historical event sequences, the ability to explore potential future climate conditions in reference to those past events, and the production of a wide variety of hourly variables.

**Fig. 1:**
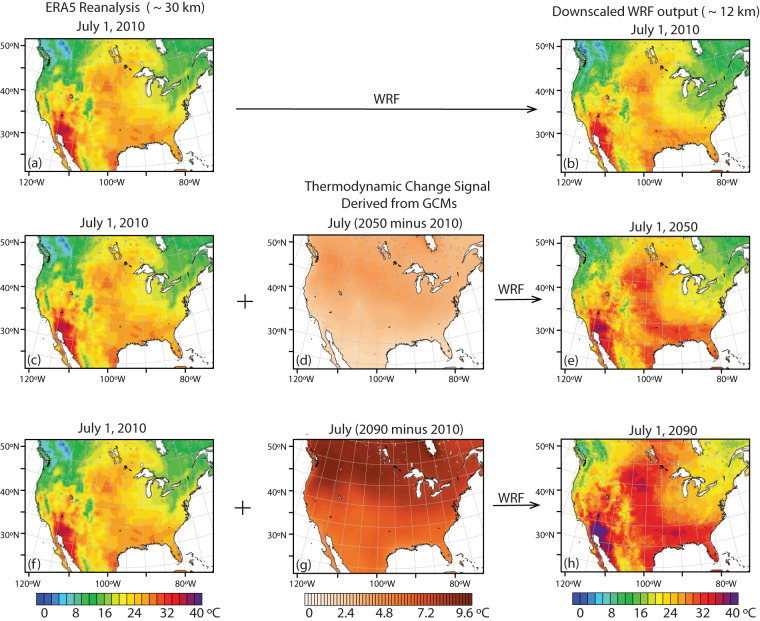
Spatial plots summarising the downscaling approach using a heat wave on July 1, 2010 as an example. Each plot shows near surface air temperature. ERA5 re-analysis (**a,c,f**) is first downscaled with the Weather Research and Forecasting (WRF) model (**b**) from a horizontal resolution of approximately 30 km to 12 km. A thermodynamic change signal derived from global climate models (GCMs) is added to the ERA5 data (d,g), representing a change from the baseline time period to 40 years in the future (July 2050 minus 2010) (**d**) and 80 years in the future (July 2090 minus 2010) (**g**). The modified ERA5 data are then downscaled with WRF yielding simulations that represent the same event from July 1, 2010 in the context of the July 1, 2050 climate (**e**) and July 1, 2090 climate (**h**). This is repeated for each time period from 1980–2019 and for 4 future warming trajectories. For illustrative purposes we show the results from the SSP585, high-sensitivity model trajectory.

While less spatially resolved, our approach shares several similarities with Liu *et al*.^35^ in that we apply the TGW approach across the entire CONUS for several years of historical reference data. Rather than shifting the entire historical period to a single future reference period, though, we derive time-evolving thermodynamic climate change signals that are applied differentially to each historical year. This allows us to produce evolving past-to-future simulations that follow a particular future warming trajectory. We begin by downscaling the ERA5 re-analysis dataset^40^ for 40 years spanning 1980–2019. We then repeat this sequence twice with added time-evolving thermodynamic warming signals that correspond to the years 2020–2059 and 2060–2099, respectively. Thus, the year 1980 is thermodynamically modified twice to represent the years 2020 and 2060. The year 1981 is modified to represent 2021 and 2061, and so on (see Fig. 1). To sample the range of possible future warming resulting from both GCM model uncertainty and emission scenario uncertainty, we do this 4 times for 4 distinct future warming trajectories: low-sensitivity models for SSP245, high-sensitivity models for SSP245, low-sensitivity models for SSP585, and high-sensitivity models for SSP585.

**Fig. 2:**
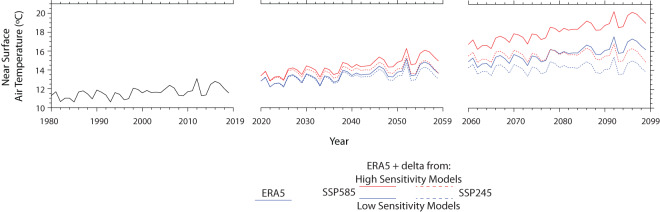
Line plots showing mean annual temperature over CONUS land area derived from ERA5 in historical period (1980–2019) and the TGW WRF simulations for high sensitivity and low sensitivity models from SSP585 (2020–2099) and SSP245 (2020–2099) in the future period. Each of the four future trajectories is composed of two 40-year sequences that repeat the historical 40-year simulation with differing levels of time-evolving thermodynamic warming signals applied.

The resulting dataset, which contains 25 hourly and over 200 3-hourly variables at 12 km spatial resolution, can be used to track a plausible range of future climate conditions across 4 future 80–year warming trajectories. Each of these 80-year trajectories is composed of two 40-year future simulations (2020–2059 and 2060–2099) that are thermodynamically modified analogies of a single 40-year historical baseline that reproduces observed historical weather events (Fig. 2). Thus, each event in the historical period is repeated 8 times (twice for each of 4 future projections) at different warming levels depending on which historical year the event occurred in. This provides a rich dataset for examining the thermodynamic implications of climate change on previously observed weather, facilitating the comparison of multiple events of a given type with one another and their analogues at multiple future warming levels.

We expect that this dataset will be widely useful for conducting climate impacts and adaptation research, including for multisector dynamics research^51^ that considers interacting impacts and adaptive responses across multiple sectors and scales. In particular, the reproduction of historical events is useful for validating the performance of downstream infrastructure models and serves as a starting point for storyline-driven research^44^. Continuously evolving projections that follow the SSP scenarios provide a foundation for examining path-dependent adaptive dynamics that interact with broader societal trends embedded in the SSPs such as population and economic growth^3^. Having a consistent baseline across all of the projections facilitates straightforward comparison of the changes associated with each future trajectory. Providing a large number of physically consistent variables at high temporal resolution enables exploration of compound events^52^ and multiple process interactions, such as hydroclimatic priming for wildfires, which involves interactions among temperature, humidity, and wind^53^. Finally, applying TGW to many events, to various types of events, and for many warming levels enables a systematic exploration of the ways in which thermodynamic change influences the characteristics of extremes.

We caution, however, that by design the TGW approach does not fully characterise the changing frequency of extreme events, since it holds internal climate variability^54^ fixed in relation to observed historical events. By the same token, our approach does not explore all possible weather patterns and events since it uses the observational record as a starting point. For example, if a tropical cyclone did not strike a given location in the historical record, it is unlikely to do so in the future TGW simulations, even if such an event could have occurred or might still occur in the future. The so-called “large ensemble” experiments^55^ represent a complementary approach that is well-suited for characterising a broad range of outcomes possible within a given climate regime, as well as changes in event frequency, or the frequency of large-scale conditions that lead to extreme events, across different climate regimes. The TGW approach captures only those aspects of event distributions that are driven by thermodynamic change, e.g., increased heat wave occurrence resulting from the intensification of events that might otherwise not have been considered heat waves. Moreover, we caution that while our approach produces a single consistent historical baseline, this historical simulation does contain biases when evaluated against observational benchmarks as we document further below. Furthermore, due to computational constraints, we limited our simulations to 12-km resolution. With sufficient resources, a similar process could be repeated at convection-resolving scales, as in Liu *et al*.^35^, which would likely improve the representation of storm dynamics and topographically driven precipitation^56^.

Finally, we note that our procedure does not result in a perfectly smooth transition from the historical period to future periods (2019 to 2020), nor from the first half of each future trajectory to its second half (2059 to 2060). The discontinuities at these transition points reflect the transition back to 1980 as the reference year at the start of each 40-year continuous simulation (both 2020 and 2060 are simulated as analogues of 1980 and are the start of a new 40-year sequence), resulting in an abrupt shift to different weather conditions and different modes of large-scale variability, such as the El Niño Southern Oscillation (ENSO). The transitions also reflect climatic differences in the warming trend from 1980–2019 between ERA5 and the GCMs used to derive the TGW signals. To assist users in understanding these transitions, we provide further analysis and discussion in the Usage Notes section below. Moreover, for some applications temporal continuity is not required and it may be preferable to analyse the data using regional or global warming levels rather than time as the primary unit of analysis. We provide examples and ancillary data about warming levels to assist users in making these determinations as well.

## Methods

### Methods overview

We downscale the European Centre for Medium-Range Weather Forecasts version 5 re-analysis (ERA5)^40^ from a spatial resolution of approximately 30 km to 12 km using the Weather Research Forecasting Model (WRF)^57^ over a 40 year period (1980–2019). For future simulations, we apply a thermodynamic global warming (TGW) approach that involves adding a climate change signal from GCMs followed by downscaling. We perform a total of four future simulations (2020–2099) for two CMIP6 scenarios and two groups of GCMs with high and low climate sensitivities, respectively. Within each group of GCMs, each model is weighted equally, and we use multiple ensemble members for each model, when available, to create a single model average before creating the multi-model average. The future simulations are divided into two forty-year simulations, 2020–2059 (near-future) and 2060–2099 (far-future). The experiments are designed such that the 40-year ERA5 historical time series repeats in the future with the added climate change signal in near and far-future simulations.

### Model selection

We select GCMs from the CMIP6 archives based on their overall skill, data availability and model independence. First, we choose GCMs that rank within the top 25 models based on a comprehensive skill assessment over the CONUS^58^. Additionally, we select the GCMs that have the data available for all the variables (air temperature, near-surface air temperature, skin temperature, relative humidity, sea-surface temperature) that are used to calculate TGW signals, for both SSP245 and SSP585 scenarios. We use 1–5 ensemble members per model based on the availability. Finally, we pick one model per institute to account for model independence, given the models from the same institute tend to perform similarly.

**Fig. 3:**
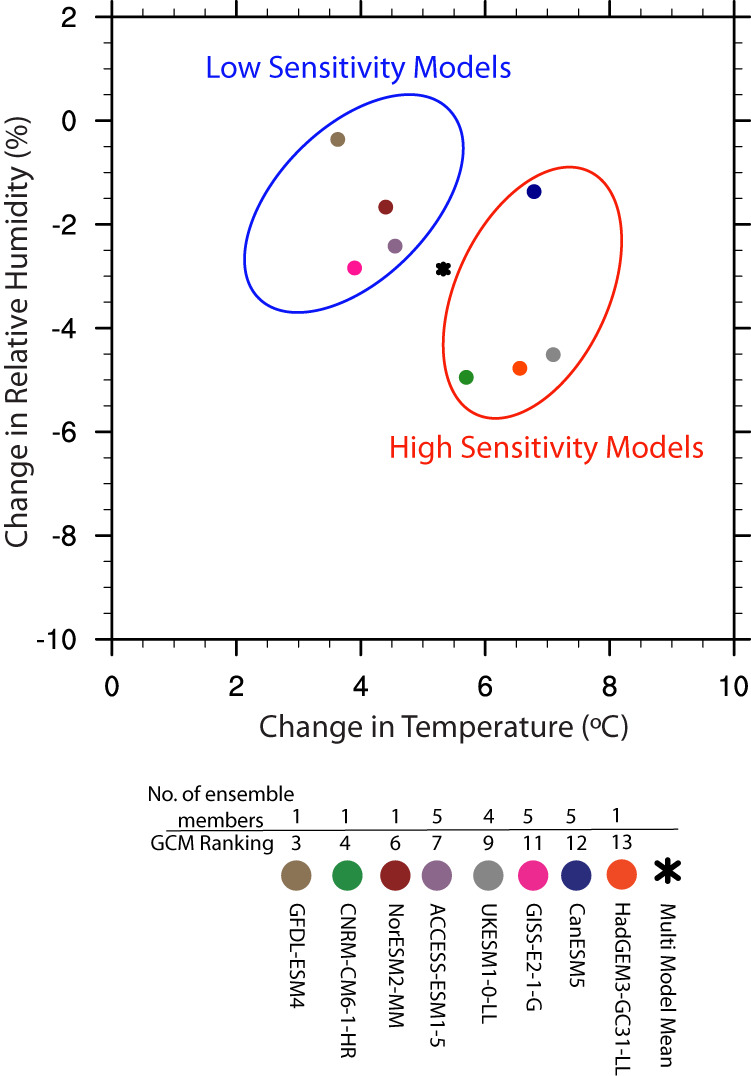
Scatter plot showing the change in temperature versus change in relative humidity over CONUS for the late 21^st^ century under SSP585 scenario with respect to historical period. The change is calculated as the difference between 2060 to 2099 and 1980 to 2019 in the ensemble mean of each of the eight CMIP6 models. Arithmetic means are used for all variables to average across time, space, and ensemble members. Models with high/low temperature sensitivity are shown in red /blue oval.

The selected models and ensembles used are summarized in Table 1. The eight selected models were further grouped into four high- and four low-climate sensitivity models based on the changes in projected CONUS mean temperature by the ensemble mean in SSP585 scenarios (Fig. 3). We also report here the transient climate response (TCR) and Equilibrium Climate Sensitivity (ECS)^59^ associated with each model, where TCR is defined as the mean global warming reached when carbon dioxide doubles in an idealised simulation in which carbon dioxide increases 1% per year, and ECS is defined as the long-term equilibrium global mean temperature change following a doubling of pre-industrial carbon dioxide concentrations. TCR has been suggested as a useful metric for identifying GCMs that exhibit realistic historical warming trajectories^60^. Each of the four low-sensitivity models exhibit a TCR within the “very likely” range of 1.2–2.4 °C as determined by the Intergovernmental Panel on Climate Change (IPCC) through multiple lines of evidence^61^. The overall mean TCR among the low-sensitivity models is 1.7 °C, which is just below the IPCC central value estimate of 1.8 °C. All of the high-sensitivity models have a TCR above the “very likely” range, with an overall mean of 2.6 °C. Considering ECS, all of the low-sensitivity models exhibit ECS values within the IPCC “very likely” range of 2.0–5.0 °C that considers multiple lines of evidence^61^, whereas 3 of the 4 high-sensitivity models exhibit ECS values above this range. The mean ECS among the low-sensitivity models is 2.92 which corresponds closely to the IPCC central estimate of 3 °C.

**Table 1:** CMIP6 models and ensemble members used to calculate thermodynamic global warming signals. The table also shows which models were designated as low-sensitivity and which were designated as high-sensitivity, along with their associated transient climate responses and equilibrium climate sensitivities. The models are ordered according to their equilibrium climate sensitivity from lowest to highest.

Model	Ensemble Members	Climate Sensitivity	Transient Climate Response (°C)	Equilibrium Climate Sensitivity (°C)
NorESM2-MM	r1i1p1f1	Low	1.22	2.49
GISS-E2-1-G	r1i1p3f1 r2i1p3f1 r3i1p3f1 r4i1p3f1 r5i1p3f1	Low	1.80	2.64
GFDL-ESM4	r1i1p1f1	Low	1.63	2.65
ACCESS-ESM1-5	r1i1p1f1 r2i1p1f1 r3i1p1f1 r4i1p1f1 r5i1p1f1	Low	1.97	3.88
CNRM-CM6-1-HR	r1i1p1f2	High	2.46	4.34
UKESM1-0-LL	r1i1p1f2 r2i1p1f2 r3i1p1f2 r4i1p1f2	High	2.77	5.36
HadGEM3-GC31-LL	r1i1p1f3	High	2.49	5.55
CanESM5	r1i1p1f1 r2i1p1f1 r3i1p1f1 r4i1p1f1 r5i1p1f1	High	2.71	5.64

### Calculation of thermodynamic global warming signals

Thermodynamic change signals were calculated using monthly GCM data for air temperature, near-surface air temperature, skin temperature, relative humidity, and sea-surface temperature. We note that some TGW studies apply change signals to temperature alone and hold relative humidity fixed. In this case, saturation vapor pressure and vapor pressure both increase according to the Clausius-Clapeyron relationship. However, as shown in Fig. 3, GCMs demonstrate changes in relative humidity that deviate from this relationship within our study domain. To capture this effect, we apply a TGW signal to relative humidity in the same way that we do for other variables, following the approach taken by other published studies^33,36^. Applying a change to both temperature and relative humidity in this way, leads to changes in other moisture related variables that are derived from those quantities by the WRF model such as vapor pressure, specific humidity, dewpoint temperature, etc. We are not aware of any studies that have systematically compared the implications of modifying relative humidity in this way versus modifying these other moisture variables directly or holding relative humidity fixed.

For each GCM, we used data from the CMIP6 historical experiment for time periods 2014 and earlier, and data from the SSP245 and SSP585 scenarios for time periods 2015 and later. Our goal was to produce a relatively smoothly evolving signal in both time and space that reflects the thermodynamic signal of climate change, but not weather-related or other modes of internal variability, since our goal is to reproduce such variability based on re-analysis. To do this, we averaged the TGW signal from multiple GCMs, multiple ensemble members for each GCM when available as listed in Table 1, and multiple years from each GCM centred on years of interest, as described below.

First, each ensemble member of a GCM was remapped to a common 1-degree latitude-longitude grid using bilinear interpolation. Additionally, the atmospheric variables (air temperature and relative humidity) were interpolated to ERA5 atmospheric pressure levels. Ensemble mean values for each GCM were created using the ensembles listed in Table 1. The multi-model mean for low and high sensitivity models was then calculated based on an average of the ensemble means.

A moving average was calculated for each month of each year from 1979 to 2099 using a 11-year window centered on the month of interest (e.g., the value for January of 1980 is the average of all Januaries from 1975–1985) to reduce the impact of interannual variability on the warming signals. Since the GCM data is available only until 2100, the size of the averaging window was reduced to 9,7,5, and 3 years as we approached 2099 (i.e., the value for January 2099 is the average of all Januaries from 2098–2100). Monthly 40-year and 80-year change signals were then calculated for each year in the 2019–2059 and 2059–2099 period with respect to the corresponding year in 1979–2019. We note that the first year of each sequence (1979, 2019, and 2059) was included to provide a spinup year for the WRF simulations; these spinup year simulations are provided separately.

The 1-degree monthly change signals were interpolated to the 12-km WRF domain. Finally, the monthly change signals were interpolated to 3-hourly levels using linear interpolation between each month. The 3-hourly 12-km signals were then added to the 3-hourly meteorological files, which were generated using the WRF pre-processing System (WPS) using the ERA5 re-analysis as input. Consequently, the modified WPS output files were used as an input to WRF to perform the future simulations. For users who wish to reproduce this workflow, the code used to calculate, interpolate, and add the TGW signals is publicly available (see Code Availability section).

### WRF configuration and testing

**Fig. 4:**
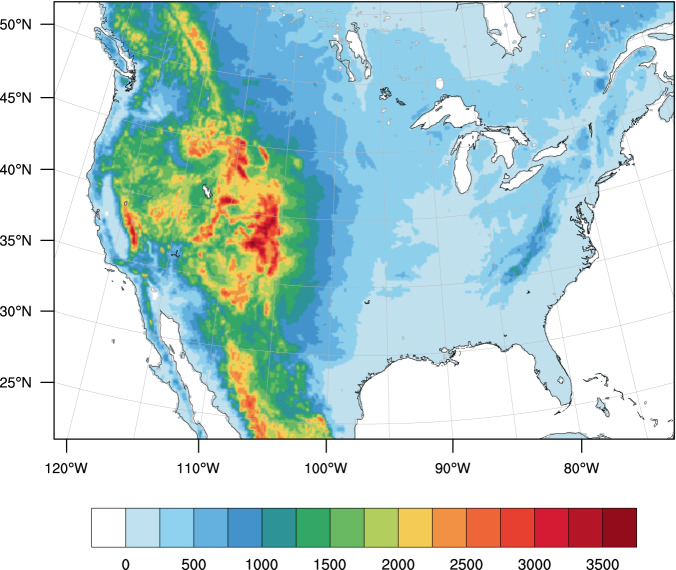
The WRF domain, which includes the CONUS, northern Mexico, and southern Canada. The colors indicate terrain height (m).

We use the Weather Research and Forecasting (WRF) model (Version 4.2.1)^57^ to downscale ERA5 in the historical period and ERA5 plus TGW signal for the future periods to a resolution of 12 km over domain size of 425 × 300 grid points over CONUS (Fig. 4). WRF is a fully compressible, non-hydrostatic, mesoscale numerical weather prediction model. WRF software architecture allows for a multitude of configurations of physics parameterizations (e.g., micro-physics, planetary boundary layer, radiation, and land surface processes) supporting a wide range of applications.

Our WRF parameterization configuration follows recommendations by the National Center for Atmospheric Research (NCAR), which is a developer and a user of the WRF model for operational forecasting over the CONUS. These recommendations have been implemented in the form of a physical parameterization suite specific to the CONUS-scale application of WRF, introduced in version 3.9. We tested two alternative parametrization configurations informed by precedents in the literature^35,56^ (Table 2) neither of which resulted in decisively improved model performance when evaluated against CONUS-level PRISM-based precipitation and daily maximum and minimum air temperature for a test simulation of the year 2009.

**Table 2:** Tested WRF physics parametrization configurations. The CONUS physics suite, recommended by NCAR, was chosen for better reproduction of the PRISM-based precipitation and daily maximum and minimum air temperature for an arbitrarily test year, 2009.

	CONUS physics suite	Rahimi *et al*.^56^	Liu *et al*.^35^
Microphysics	Thompson scheme^88^	Predicted Particle Property scheme (P3)^89^	Thompson aerosol-aware^90^
Cumulus Parameterization	Tiedtke^91^	Tiedtke^91^	—
Longwave Radiation	Rapid Radiative Transfer Model^92^	Rapid Radiative Transfer Model^92^	Rapid Radiative Transfer Model^92^
Shortwave Radiation	Rapid Radiative Transfer Model^92^	Rapid Radiative Transfer Model^92^	Rapid Radiative Transfer Model^92^
Planetary Boundary layer	Mellor-Yamada-Janjic scheme (MYJ)^93,94^	Yonsei University PBL (YSU)^95^	Yonsei University PBL (YSU)^95^
Surface Layer	Eta Similarity scheme^96^	Fifth-Generation Penn State/NCAR Mesoscale Model (Revised MM5)^97^	Fifth-Generation Penn State/NCAR Mesoscale Model (Revised MM5)^97^
Land Surface	Noah Land Surface Model^62^	Noah-MP^98^	Noah-MP^98^

For all simulations, we used the Noah Land Surface Model^62^, the National Land Cover Data (NLCD)^63^, the NLCD impervious surface data^64^, and the single-layer urban canopy model (UCM)^65,66^ for an enhanced representation of urban cover (fraction of developed or built surfaces) and processes. The Noah Land Surface model was chosen due to its compatibility with UCM and NLCD. UCM resolves urban canopy processes and the exchange of energy, moisture, and momentum between urban surfaces and the planetary boundary layer. The UCM parametrizes the three-dimensional nature of the street canyon where it accounts for the shadowing, reflection, and trapping of the radiation and wind profiles^67^. Representation of urban surfaces with adequate fidelity is important for two reasons. First, urban areas are increasingly shown to have important implications for atmospheric moisture, wind, boundary layer structure, cloud formation, precipitation, and storms^68^. Second, surface observations, especially over urban areas, are rarely assimilated in re-analysis datasets^68,69^, including in ERA5.

All simulations use 39 vertical levels and daily sea-surface temperature (SST). Following reports of improved model performance and to prevent significant model drift over long simulations^35,56,70,71^, we apply spectral nudging to the temperature, geopotential height, and horizontal winds. Nudging starts from the planetary boundary layer (PBL) top, an approach well-established in the literature^35,56,72,73^, up to the model top of 50 hPa. For spectral nudging, the zonal and meridional grid-relative wavenumber parameters, were set to 3 which constrains large scale phenomena (larger than ~1,500 km) while allowing for mesoscales and sub-synoptic scales such as convective systems to evolve freely. Atmospheric concentrations of the principal greenhouse gases (CO_2_, N_2_O, CH_4_, CFC11, and CFC12) within WRF are specified to follow the SSP scenarios for CMIP6 experiment^74^ in a time-evolving annual manner. Land use and landcover do not change over time. Each 40-year simulation begins with an additional spinup year (e.g., we simulate 1979 prior to the 1980–2019 simulation) in order to allow the model’s internal state variables to approach equilibrium before proceeding with the target simulation years. We note that soil moisture states typically take several years to equilibrate following a discontinuous change in climate regime. Therefore, users should be aware there may be transitory soil moisture and associated land-atmosphere feedback dynamics in the first few years of each 40-year simulation. See the Usage Notes section for further discussion of discontinuities among the simulations.

## Data Records

The dataset^75^ is available via Globus with a copy stored and minted in the MSD-Live data repository (10.57931/1885756). Instructions for downloading the data, as well as additional information regarding the dataset and available variables can be found at a data landing page (https://tgw-data.msdlive.org).

The dataset consists of WRF model outputs for each of 9 40-year simulations (see Table 3), yearly restart files for each simulation that can be used to restart the WRF model, and the average warming signal applied to each trajectory. The aforementioned spinup years of each simulation are provided in a separate folder, but are not recommended for use beyond diagnostic purposes.

**Table 3:** A list of simulations indicating the corresponding SSP scenario, RCP pathway, GCM sensitivity designation, and time period. The folder name for locating data associated with each simulation within the data repository is provided as well.

Simulation	SSP	RCP	GCM Sensitivity	Time Period	Folder Name
HISTORICAL	—	—	—	1980–2019	historical_1980_2019
SSP245COLD_NEAR	2	4.5	Low	2020–2059	rcp45cooler_2020_2059
SSP245COLD_FAR	2	4.5	Low	2060–2099	rcp45cooler_2060_2099
SSP245HOT_NEAR	2	4.5	High	2020–2059	rcp45hotter_2020_2059
SSP245HOT_FAR	2	4.5	High	2060–2099	rcp45hotter_2060_2099
SSP585COLD_NEAR	5	8.5	Low	2020–2059	rcp85cooler_2020_2059
SSP585COLD_FAR	5	8.5	Low	2060–2099	rcp85cooler_2060_2099
SSP585HOT_NEAR	5	8.5	High	2020–2059	rcp85hotter_2020_2059
SSP585HOT_FAR	5	8.5	High	2060–2099	rcp85hotter_2060_2099

Data for each simulation are provided in weekly NetCDF files that include additional metadata such as units, descriptions, and dimensionality for each variable. 25 variables are available at hourly resolution, and 207 variables are available at three-hourly resolution. Spatial resolution is 12 km and spans CONUS, including some areas of Canada and Mexico, resulting in a grid of 424 by 299 cells. Certain variables are also stratified across 32 pressure levels or 4 soil layers. The spatial projection is a Lambert Conformal Conic with a reference latitude of 40°, a reference longitude of −97°, standard parallels of 30° and 45°, and using an Earth radius of 6.37 × 10^6^m. Spatial coordinates are provided in both meters and degrees. For a full list of variables, please see the dataset landing page on the MSD-LIVE platform.

To aid in the contextualization of each simulated future year, spatially averaged warming signals are provided in degrees Celsius as CSV files at both monthly and annual temporal resolution, at both global and CONUS scale. These represent the degree of overall warming applied to each historical reference time period to produce the future simulations and can be used to translate from a time-based analysis framework to a warming level-based framework as described in the usage notes section below.

## Technical Validation

Similar to GCMs, regional climate models are known to exhibit biases when compared against historical data^35,56^. It is therefore important to understand the extent of such biases so that potential users of the data can determine suitability for specific applications and interpret results accordingly. Here we evaluate the extent of the biases present in our historical simulation dataset for common metrics related to temperature and precipitation. We focus our evaluation across a range of spatial and temporal scales including seasonal means as well as metrics related to environmental extremes and distributions at specific points. It is not possible to anticipate and evaluate all of the climatic phenomena of potential interest to users of the data, so we encourage users to perform additional validation of the historical data for specific applications as appropriate. To illustrate how one might perform additional validation with respect to a specific event type, we provide an analysis of tropical cyclone tracks and intensities.

Because our historical simulation is a downscaling of the ERA5 re-analysis dataset, some of the biases may be inherited from that parent dataset and some may be introduced by WRF. We therefore present, where possible, evaluations that compare our historical simulation directly to ERA5, as well as to benchmark observationally based datasets, which helps to decompose which biases arise from ERA5 versus WRF.

### Temperature and precipitation

**Fig. 5:**
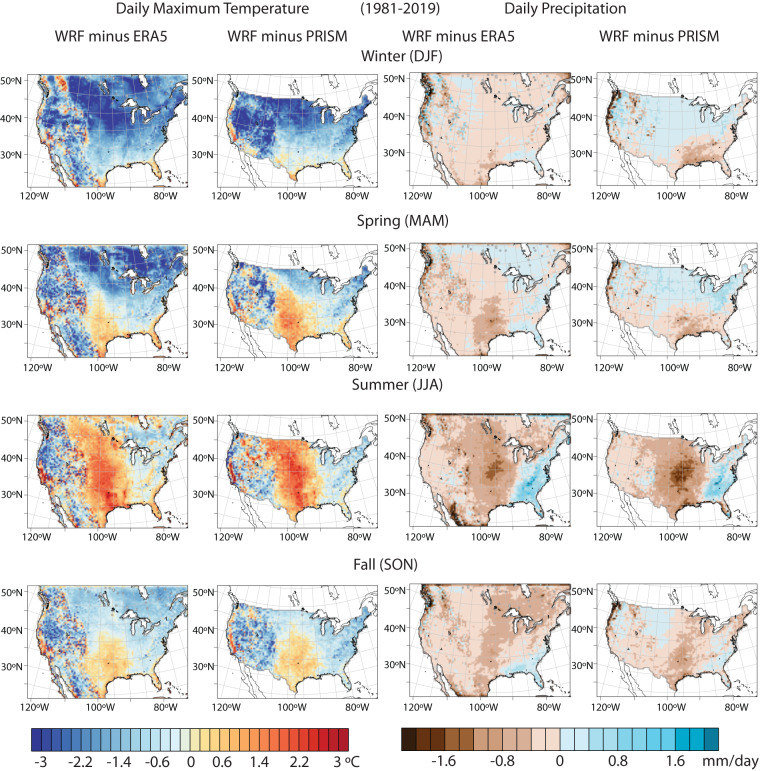
Spatial maps showing differences in climatology of daily maximum temperature and precipitation simulated by WRF with respect to ERA5 and PRISM for (**a**–**d**) Winter (**e**–**h**) Spring (**i**–**l**) Summer and (**m**–**p**) Fall during the 1981–2019 period.

We evaluate the spatial pattern of seasonal mean daily maximum temperature and precipitation in our historical dataset compared to the ERA5 re-analysis dataset and to the observationally derived Parameter-elevation Regressions on Independent Slopes Model (PRISM)^76^ dataset (Fig. 5; Table 4). Because PRISM data is not available for 1980, we restrict this and subsequent analyses to the common overlapping years of 1981–2019.

**Table 4:** Seasonal bias statistics comparing the WRF historical simulation during the period 1981–2019 to both the ERA5 and PRISM datasets, including CONUS-wide mean absolute biases for daily maximum temperature and precipitation, as well as the spatial pattern correlation coefficients mean daily maximum temperature and precipitation across the CONUS.

	Mean Absolute Daily Maximum Temperature Bias (°C)		Mean Absolute Daily Precipitation Bias (mm)	
	WRF vs. ERA5	WRF vs. PRISM	WRF vs. ERA5	WRF vs. PRISM
Winter (DJF)	1.73	1.68	0.24	0.33
Spring (MAM)	1.12	0.97	0.31	0.30
Summer (JJA)	1.09	1.04	0.56	0.58
Fall (SON)	0.78	0.81	0.36	0.32
	**Mean Daily Maximum Temperature Spatial Pattern Correlation**		**Mean Daily Precipitation Spatial Pattern Correlation**	
	**WRF vs. ERA5**	**WRF vs. PRISM**	**WRF vs. ERA5**	**WRF vs. PRISM**
Winter (DJF)	0.99	0.99	0.96	0.96
Spring (MAM)	0.99	0.99	0.95	0.94
Summer (JJA)	0.95	0.95	0.91	0.90
Fall (SON)	0.99	0.99	0.95	0.95

Overall, the spatial patterns of seasonal temperature and precipitation in the WRF historical simulation are highly correlated with both datasets. All correlation coefficients for seasonal mean daily maximum temperature are 0.95 or higher and all correlation coefficients are 0.90 or higher for seasonal mean daily precipitation (Table 4). The lowest correlation is for summertime precipitation, where a clear dry and hot bias can be seen over the central United States (Fig. 5), which is a bias identified in other studies using the WRF model^35^. There is also a cold bias during the winter season (Fig. 5). Overall, the patterns of WRF bias compared to ERA5 and PRISM are similar to one another, indicating that they arise primarily from WRF, as opposed to ERA5.

**Fig. 6:**
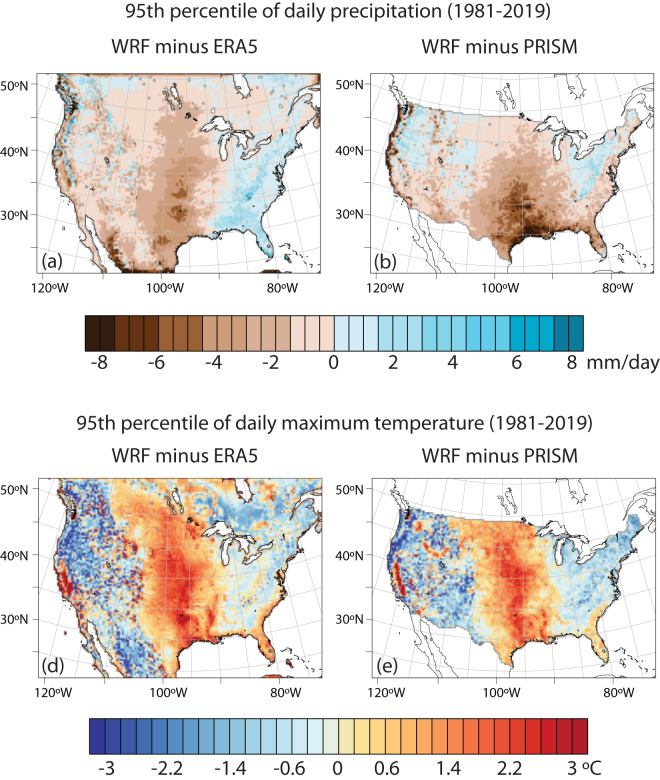
Spatial maps showing differences in climatology of 95th percentile of daily precipitation (P95) and 95th percentile of daily maximum temperature (T95) simulated by WRF with respect to (**a,d**) ERA5 and (**b,e**) PRISM.

**Fig. 7:**
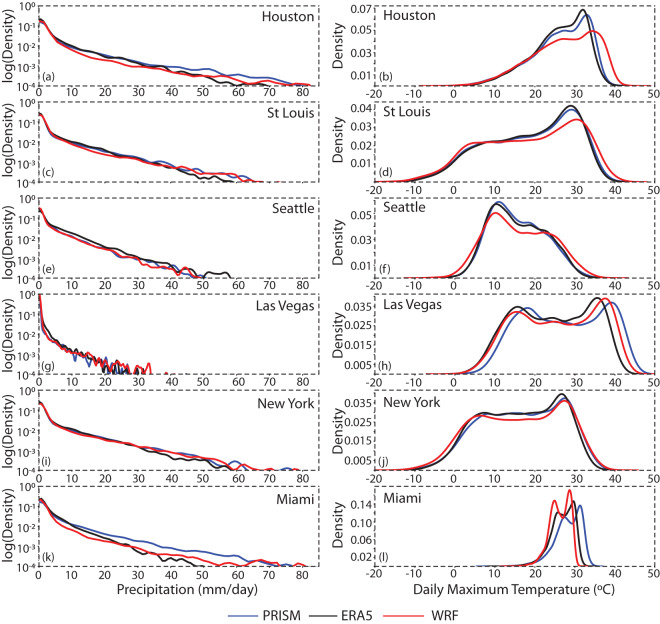
Probability distribution plots of daily precipitation and daily maximum temperature over (**a,b**) Houston, (**c,d**) St Louis (**e,f**) Seattle (**g,h**) Las Vegas (**i,j**) New York and (**k,l**) Miami respectively. These plots use daily data from 1981–2019 period. Precipitation distributions are presented with a log probability scale.

In terms of temperature and precipitation extremes, we also evaluated the spatial pattern of the 95% percentile for daily maximum temperature and precipitation (Fig. 6). This analysis reveals a hot and dry pattern over the central United States that resembles the bias for summertime seasonal mean maximum temperature and precipitation. To understand these biases further, we evaluated the shape of the distributions for daily maximum temperature and precipitation at 6 points across the CONUS (Fig. 7). These representative points all lie over major metropolitan areas and were chosen for their geographic and climatic diversity, as well as to sample points with both higher and lower levels of bias in the 95% percentile of maximum temperature and precipitation. The temperature distributions show that there is overall good correspondence between the WRF dataset, ERA5, and PRISM, with the WRF dataset often capturing unique multimodal peaks observed at each point. However, the central US points (Houston and St Louis) are skewed toward high extremes, as is the Seattle point to a lesser extent. The Las Vegas point shows an improvement over ERA5, whereas the Miami point shows a degradation toward lower extremes compared to ERA5. In several cases, the WRF dataset shows an improvement over ERA5 for lower levels of precipitation (Fig. 7). However, in regions of higher bias in the 95% percentile value such as Houston and St. Louis, the distribution has greater density in the 1–4 mm range, which corresponds to lower density at the higher end of the distribution.

### Tropical cyclone tracks and intensities

Tropical cyclones (TCs) are weather phenomena that threaten the U.S. East and Gulf Coasts in the summer and fall months and are being impacted by climate change^43,77^. Because TC landfalls cause dangerous conditions through their heavy precipitation, storm surge, and strong winds, it is important to understand how TCs and their hazards may change in future climates, and TGW simulations provide a unique testbed to study this question. Here we present the TC tracks and intensities in the WRF Historic simulation to ensure the TC climatology looks reasonably realistic and can be compared to observations and re-analysis.

TCs were tracked using the TempestExtremes package^78,79^ using parameters very similar to those described in Stansfield *et al*.^80^. For observations, the International Best Track Archive for Climate Stewardship (IBTrACS) database^81^ is used for TC tracks and intensities. TCs are also tracked in ERA5^40^ for comparison, since the WRF Historical simulation is downscaled from ERA5. One additional step we applied for the WRF simulations was discarding any TC track points that were directly on the WRF domain edge. For observations and ERA5, any track points outside of the WRF domain or on the domain edge were discarded. Since WRF has a bounded domain and therefore it is harder to satisfy TC tracking criteria within the limited domain, we applied the following tracking filters used on the WRF output to the observations and ERA5: each trajectory must last for at least 24 hours with a maximum gap of 6 hours, the maximum 10-m wind speed had to be at least 10 m/s for 2 timesteps in each trajectory, and the first latitude point of each trajectory had to be at or equatorward of 35°N.

**Fig. 8:**
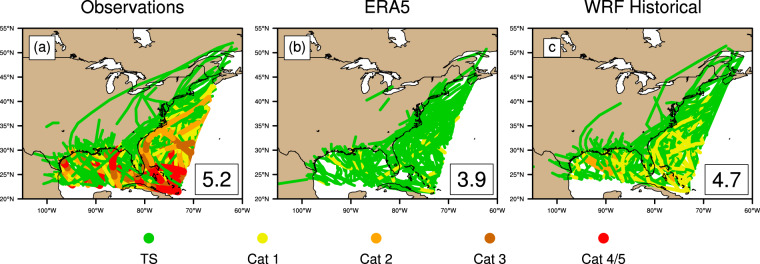
Tracks of TCs in (**a**) observations, (**b**) ERA5, and (**c**) the WRF Historical simulation for 1980–2019. Tracks are colored by their Saffir-Simpson category at the time, based on their 10-m wind speed (see legend at bottom). The number in the bottom right of each panel shows the annual mean TC count. Tracks are only plotted for times when the TC’s maximum 10-m wind speed is 17 m/s or higher.

Looking at Fig. 8, the locations of TC landfalls are similar across observations, ERA5, and WRF; however, the ERA5 and WRF Historical underestimate the annual mean TC counts over the 40-year period compared to observations. Looking at the TC intensities, WRF Historical shows improvement over ERA5, simulating many more storms at the Category 1 and 2 strengths. The TCs in WRF do not reach major hurricane strength (Category 3 and above), which is common for atmospheric models^82^ but also could be related to the short distance between the WRF domain edge and CONUS, which does not allow much time for the TCs to intensify over the ocean before making landfall. In short, the WRF Historical simulation underestimates annual mean TC counts compared to observations, like ERA5, but improves upon the low bias in annual mean TC counts and TC intensities in ERA5^80,83^. Nevertheless, it would still be fruitful for future studies to utilize these WRF simulations to understand how TCs and their dangerous characteristics like precipitation totals may change in the future.

## Usage Notes

The dataset lends itself to examining future changes in climate conditions and specific weather events in two distinct ways: either as they evolve over time in relation to specific warming trajectories (time-based analysis) or alternatively as a function of the overall amount of warming present in different future analogue simulations of past conditions (warming level-based analysis). Doing so with respect to particular events, especially events that led to significant impacts on human or natural systems, has been referred to as “storyline” analysis^44^. To assist users in translating between time-based and warming level-based analysis, the dataset includes several files that contain the mean annual and monthly surface air temperature warming levels associated with each future simulation, calculated as both CONUS mean changes and global mean changes using the same procedure and GCM groupings that were used to derive the TGW signals driving the simulations.

In this section we provide some illustrative examples of how one might use the dataset to examine storylines, as well as future climate changes with respect to both time and warming levels. When examining changes over time, it is important to be aware of discontinuities in the dataset that occur at the boundaries of each 40-year simulation (i.e., at the 2019 to 2020 transition and the 2059 to 2060 transition). Therefore, we provide some additional analysis to assist users in understanding why these discontinuities occur and to contextualise them in relation to the mean warming trajectories estimated by GCMs.

### Tropical cyclone storyline precipitation analysis

**Fig. 9:**
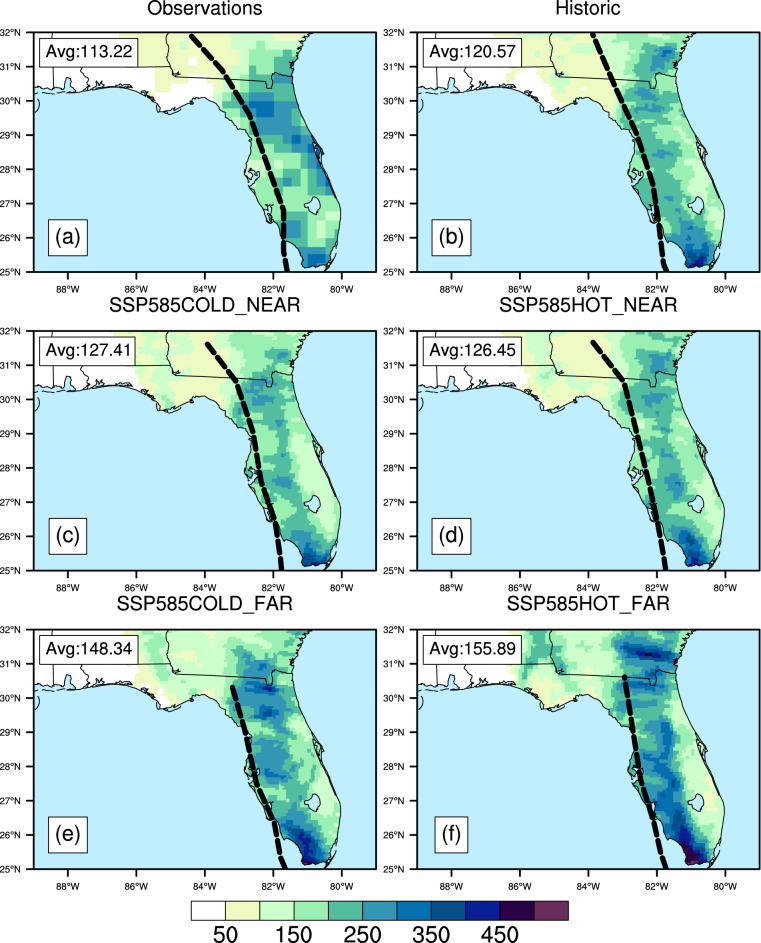
Precipitation from Hurricane Irma over Florida in (**a**) observations, (**b**) the WRF Historical simulation, and (**c**–**f**) the four SSP585 WRF simulations. Colored contours show accumulated precipitation in mm for Sept. 9 0Z 2017 to Sept. 12 0Z 2017. Black dashed lines show the track (observed or simulated) of Irma. The track terminates earlier in some simulations than others because TCs are only tracked when their maximum 10-m wind speeds meet or exceed 17 m/s. The text in the upper left shows the average accumulated precipitation over Florida only.

Figure 9 shows the accumulated precipitation in mm from Hurricane Irma over the period Sept. 9, 2017 at 0 Z to Sept. 12, 2017 at 0Z for observations, the WRF Historical simulation, and the four SSP585 WRF simulations. TC precipitation was extracted using the TempestExtremes package^78,79^ using methodology described in Stansfield *et al*.^80^ For observations, the precipitation dataset used is the CPC Global Unified Gauge-Based Analysis of Daily Precipitation^84^. As shown by the dashed black lines, the tracks of Irma are similar in the simulations compared to observations. Similar tracks are a prerequisite to compare precipitation accumulations because deviations in a TC’s track can cause changes in landfall location and translation speed, which can make it hard to directly compare the precipitation^43^. Therefore, before comparing a TC between different future simulations, it’s important to plot its track in the various simulations first and make sure they are similar, which may not be true for all TCs in the dataset. Besides comparing the spatial field of precipitation, bulk metrics like average precipitation accumulations over an area can be compared, as shown in the boxes in the top left for the state of Florida only. For Hurricane Irma, the spatial fields of accumulated precipitation are similar across all the WRF simulations, with maximum values along the western coast and central Florida. It is also obvious that the precipitation accumulations generally increase for the future simulations compared to the Historical, which is confirmed by the average accumulation values.

### Time-based and warming level-based analysis

**Fig. 10:**
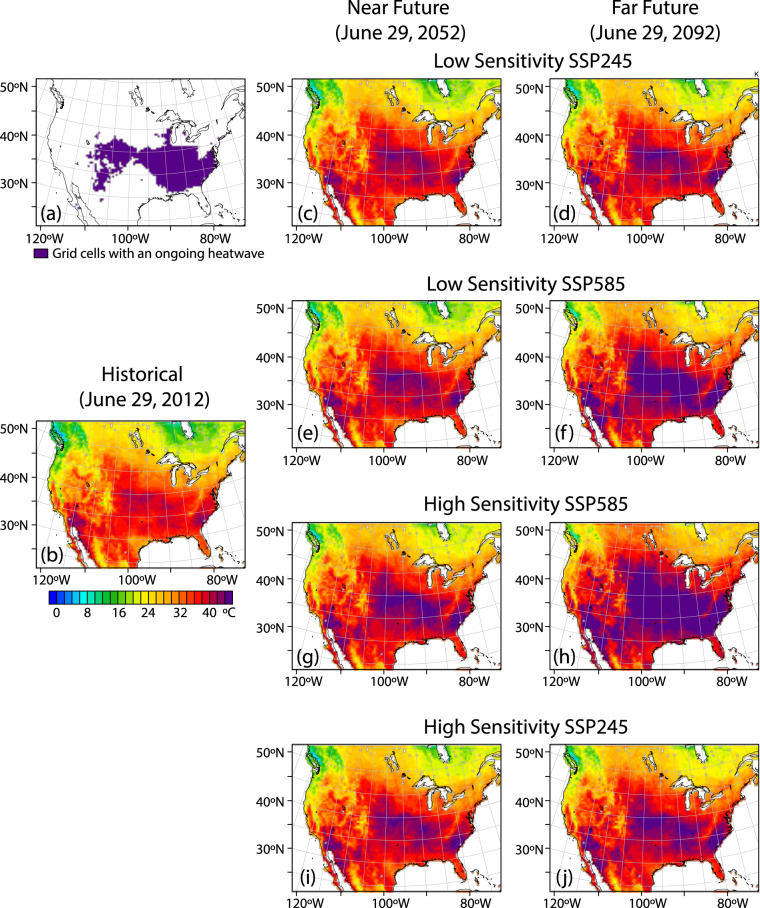
Spatial maps showing (**a**) grid points that have an ongoing heat wave and daily maximum temperature on a particular day in (**b**) historical and in near and far future periods for (**c,d**) SSP245 low sensitivity (**e,f**) SSP585 low sensitivity (**g,h**) SSP585 high sensitivity and (**i,j**) SSP245 high sensitivity simulations. The plots are shown for June 29 in the year 2012, 2052 and 2092 in historical, near and far future respectively. A heat wave at a given grid point must have at least 3 days with daily maximum temperature above a threshold. The threshold is determined by calculating the 95th percentile of daily maximum temperature during the summer months (June, July & August) of each year and then averaging this value over the 1980–2019 period.

**Fig. 11:**
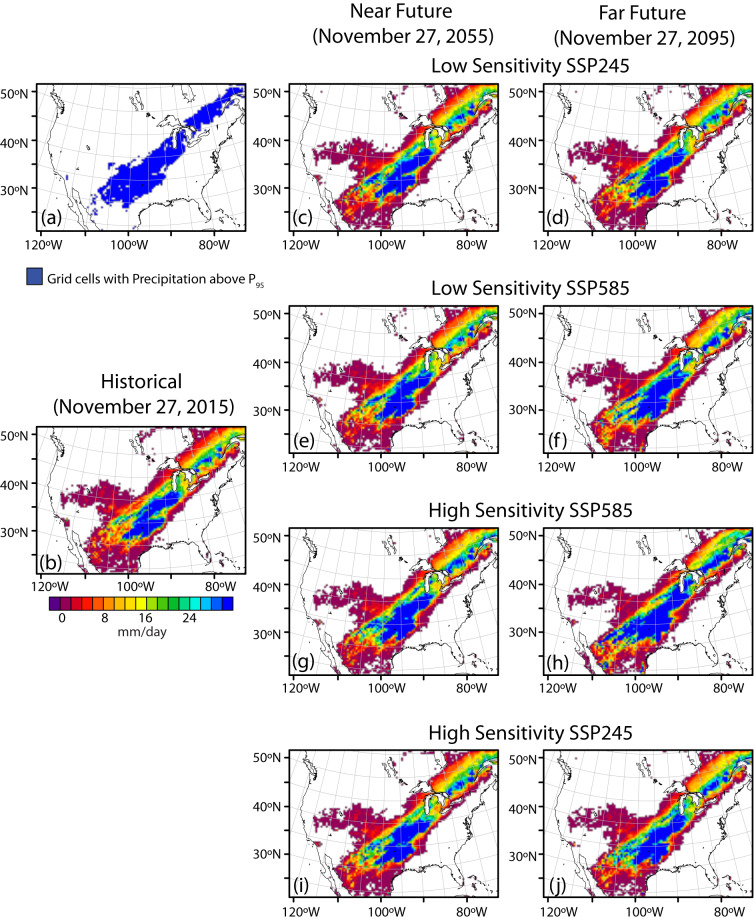
Spatial maps showing (**a**) grid points that have an ongoing extreme precipitation event and precipitation on a particular day in (**b**) historical and in near and far future periods for (**c,d**) SSP245 low sensitivity (**e,f**) SSP585 low sensitivity (**g,h**) SSP585 high sensitivity and (**i,j**) SSP245 high sensitivity simulations. The plots are shown for November 27 in the year 2015, 2055 and 2095 in historical, near and far future respectively. The grid points shown in a) are those with precipitation above a threshold, which is determined by calculating the 95th percentile of daily precipitation for each year and then averaging over the 1980–2019 period.

**Fig. 12:**
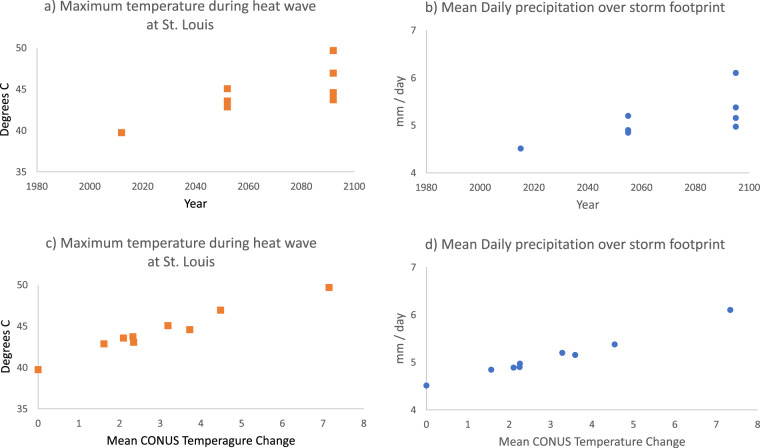
Scatter plots demonstrating the difference between time-based and warming level-based analysis of individual event characteristics. Panels (**a**) and (**c**) show the maximum temperature simulated at a single gridcell at St Louis, MO during the heat wave June 29, 2012 heat wave event and its 8 future analogue events simulated in 2052 and 2092. Panels (**b**) and (**d**) show the mean daily precipitation averaged over gridcells in which precipitation is present during the storm event that occurred on November 27, 2015 and its 8 future analogue events in 2055 and 2095. Panel (**a**) and (**b**) display this information with respect to time, whereas panels (**c**) and (**d**) display the same values with respect to the relative amount of CONUS-scale mean warming in each warming trajectory.

To demonstrate how to translate between time-based and warming level-based analysis using the dataset, we consider two illustrative events: a heat wave that occurred on June 29, 2012 (Fig. 10) and an extreme precipitation event that occurred on November 27, 2015 (Fig. 11). Each event is simulated once in the historical simulation and 8 times within the future TGW simulations, twice (at plus-40 and plus-80 years) for each of the 4 future warming trajectories. The spatial extent and maximum temperatures reached during the heat wave increase in the future simulations, more so in the later time periods compared to the earlier periods from the same trajectories, more so in the in the high-sensitivity model groups compared the low-sensitivity model groups, and more so in the SSP585 scenarios compared to the SSP245 scenarios. Similarly, the precipitation event is simulated once in the historical simulation and 8 times within the future TGW simulations, and generally shows higher levels of precipitation in those time periods and trajectories that have higher amounts of warming, such as later time periods, high-sensitivity model groups, and higher emissions scenarios. To further examine the relationship between warming level and event characteristics, we chose an illustrative scalar metric for each event that describes a key characteristic of the event. For the heat wave, we chose the maximum temperature reached at a specific gridcell near the center of the heat wave over the city of St. Louis. For the precipitation event, we calculate the mean daily precipitation over all gridcells within the CONUS where precipitation occurs. The characteristics can be understood in relation to the warming trajectories over time (Fig. 12, panels a and b), where the future values at each time represent a range of plausible changes given the GCM sensitivities and emission scenarios considered. Alternatively, the characteristics can be evaluated in relation to the amount of regional warming applied to each TGW simulation (Fig. 12, panels c.d), which highlights the degree to which varying levels of thermodynamic change influence the characteristics of the events.

### Transitions among the simulations

Each 40-year simulation in the dataset evolves continuously and can be used to track individual weather events. However, discontinuities occur at the boundary between each 40-year simulation (from 2019 to 2020 and from 2059 to 2060). These discontinuities result from two factors. First, there is a shift in which reference year is used as the baseline. For instance, 2019 is a direct downscaling of ERA5 for the year 2019, but 2020 is simulated as a thermodynamically modified version of 1980. Likewise, 2059 is a thermodynamically modified version of 2019, but 2060 is a thermodynamically modified version of 1980. This means that there is a transition to a new weather pattern and new large-scale modes of variability (e.g., a new degree of strength in the El Nino Southern Oscillation) that occur at those transition points. Therefore, users should be mindful not to treat any particular event as continuous across those transitions.

**Fig. 13:**
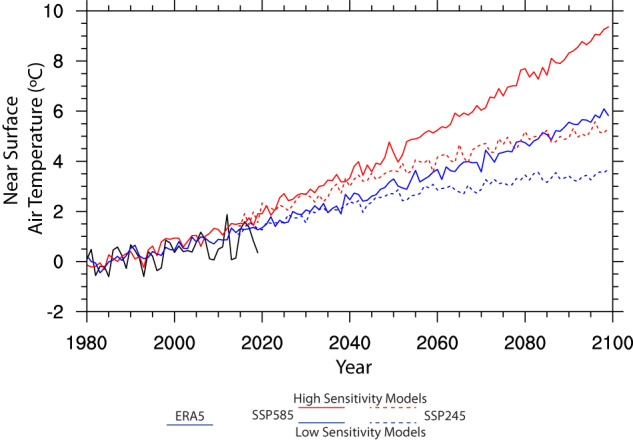
Mean annual surface air temperature anomalies over the CONUS in ERA5 and multi-model GCM means for each of the four warming trajectories. Each trajectory is normalised relative to its own 1980–1989 mean temperature in order to facilitate comparison of their relative warming rates over time.

**Fig. 14:**
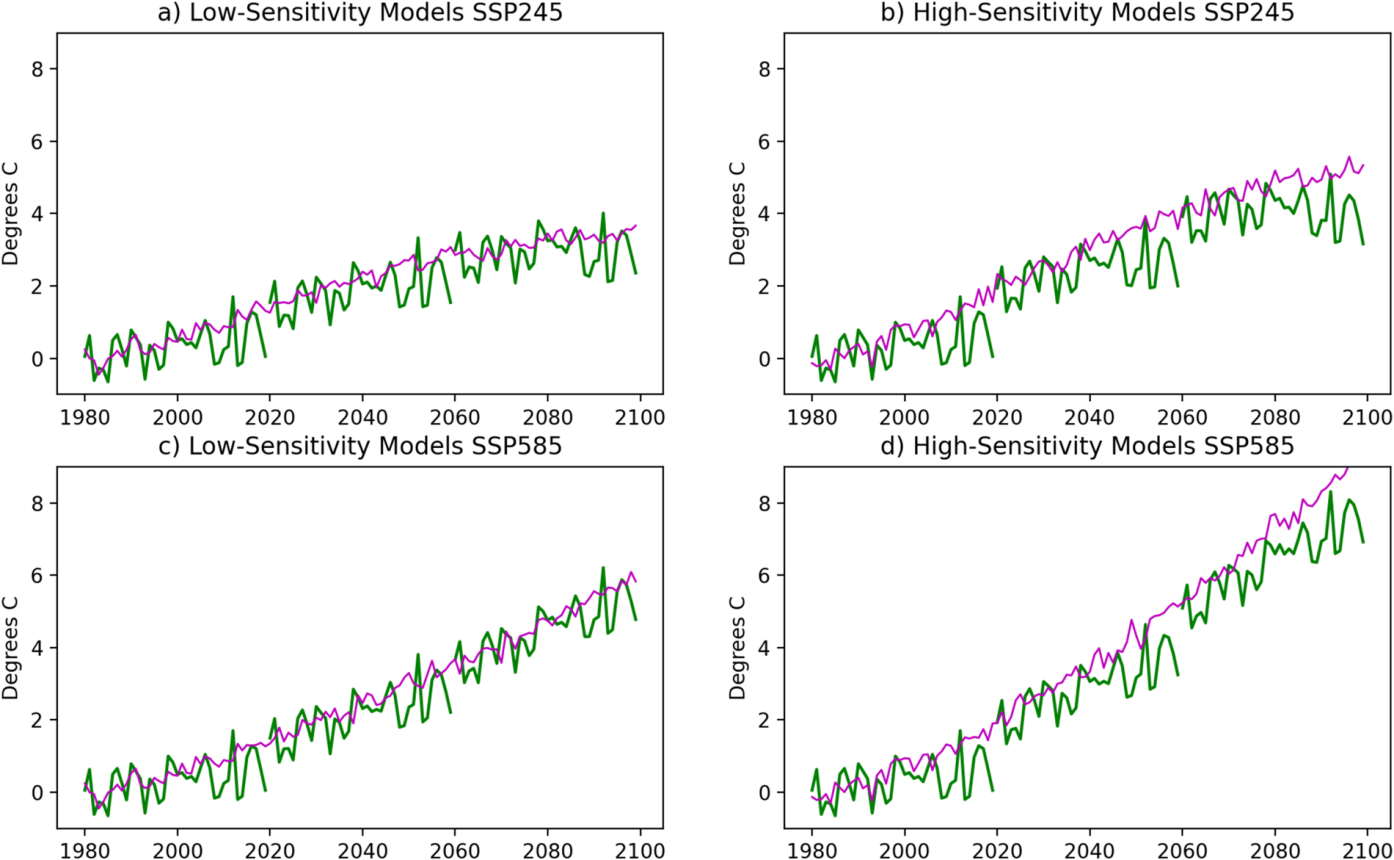
Mean annual surface air temperature anomalies over the CONUS in the WRF TGW simulations (green) and multi-model GCM means for each of the four warming trajectories (magenta): (**a**) low-sensitivity models for SSP245, (**b**) high-sensitivity models for SSP245, (**c**) low-sensitivity models for SSP585, and (**d**) high-sensitivity models for SSP585. Each trajectory is normalised relative to its own 1980–1989 mean temperature in order to facilitate comparison of their relative warming rates over time.

The second factor contributing to discontinuity among the separate 40-year simulations results from a mismatch between the amount of warming that occurs within ERA5 during the 1980–2019 historical baseline period in comparison to the GCMs used to derive the TGW signals (Fig. 13). In general, the GCMs warm at a faster rate than ERA5. This is especially true for the higher sensitivity model warming trajectories. Therefore, the TGW warming signal for the year 2020, which is based on a multi-year and multi-model average representing the GCM change from 1980 to 2020, exceeds the amount of warming present in ERA5 during that same time period. This results in a discontinuity within the WRF TGW dataset at 2020 as the WRF simulations essentially catch up to the level of warming present in the GCMs in 2020 (Fig. 14). Over the course of the 2020–2059 simulations, the lower level of warming in the baseline ERA5 dataset causes the downscaled WRF TGW simulations to eventually track below the GCM warming trajectories, which again results in a discontinuity at 2060 as the simulations return to 1980 as the baseline year and the TGW WRF simulation again catches up to the GCM trajectory.

As can be seen in Fig. 14, the WRF TGW simulations broadly track the GCM warming trajectories, albeit in a piecewise manner. The magnitude of the transition discontinuities is more prevalent in the high-sensitivity model simulations because the difference in the baseline warming level in this GCM group compared to ERA5 is larger than that in the low-sensitivity GCM model group. Indeed, as noted in the methods section above, the low-sensitivity model group contains GCMs whose transient climate response (TCR) and equilibrium climate sensitivity (ECS) fall within the “very likely” range based on the multiple lines of evidence assessed within the most recent Intergovernmental Panel on Climate Change assessment report^61^, whereas the high-sensitivity model group contains models whose TCR’s all exceed this range whose ECS’s exceed this range in 3 of 4 cases. It has been suggested that such high-sensitivity models be used with caution when examining future warming in a time-based manner^60^, although others have noted that it is not possible to exclude these high-sensitivity models as implausible and thus they retain value for exploring a plausible range of extreme conditions^85^. Similarly, there is a growing understanding that the SSP585 scenario is unlikely given physical constraints on emissions as well as current policy and economic trends^86^, yet it retains analytic value when understood as a plausible extreme case^87^ and also provides a high signal-to-noise ratio for exploring how climate change affects the characteristics of weather phenomena.

## Data Availability

Code and workflow for running the historical simulation is available at https://github.com/IMMM-SFA/jones-etal_2023_scidata. Model versions, input datasets, and parameters are outlined within.

## References

[1] Eyring V (2016). Overview of the Coupled Model Intercomparison Project Phase 6 (CMIP6) experimental design and organization. Geosci. Model Dev..

[2] O’Neill BC (2016). The Scenario Model Intercomparison Project (ScenarioMIP) for CMIP6. Geosci. Model Dev..

[3] Riahi K (2017). The Shared Socioeconomic Pathways and their energy, land use, and greenhouse gas emissions implications: An overview. Glob. Environ. Change.

[4] van Vuuren DP (2011). The representative concentration pathways: an overview. Clim. Change.

[5] Haarsma RJ (2016). High Resolution Model Intercomparison Project (HighResMIP v1.0) for CMIP6. Geosci. Model Dev..

[6] Zarzycki CM (2014). Aquaplanet Experiments Using CAM’s Variable-Resolution Dynamical Core. J. Clim..

[7] Tang Q (2023). The fully coupled regionally refined model of E3SM version 2: overview of the atmosphere, land, and river results. Geosci. Model Dev..

[8] Rhoades AM (2018). Sensitivity of Mountain Hydroclimate Simulations in Variable-Resolution CESM to Microphysics and Horizontal Resolution. J. Adv. Model. Earth Syst..

[9] Rahimi E, Barghjelveh S, Dong P (2021). Quantifying how urban landscape heterogeneity affects land surface temperature at multiple scales. J. Ecol. Environ..

[10] Pierce DW, Cayan DR, Maurer EP, Abatzoglou JT, Hegewisch KC (2015). Improved Bias Correction Techniques for Hydrological Simulations of Climate Change. J. Hydrometeorol..

[11] Teutschbein C, Seibert J (2010). Regional Climate Models for Hydrological Impact Studies at the Catchment Scale: A Review of Recent Modeling Strategies. Geogr. Compass.

[12] Hawkins E, Sutton R (2011). The potential to narrow uncertainty in projections of regional precipitation change. Clim. Dyn..

[13] Lehner F (2020). Partitioning climate projection uncertainty with multiple large ensembles and CMIP5/6. Earth Syst. Dyn..

[14] Goldenson, N. *et al*. Use-Inspired, Process-Oriented GCM Selection: Prioritizing Models for Regional Dynamical Downscaling. *Bull. Am. Meteorol. Soc*. **1** (2023).

[15] Fowler HJ, Blenkinsop S, Tebaldi C (2007). Linking climate change modelling to impacts studies: recent advances in downscaling techniques for hydrological modelling. Int. J. Climatol..

[16] Pierce DW, Cayan DR, Thrasher BL (2014). Statistical Downscaling Using Localized Constructed Analogs (LOCA). J. Hydrometeorol..

[17] Pierce DW, Cayan DR (2016). Downscaling humidity with Localized Constructed Analogs (LOCA) over the conterminous United States. Clim. Dyn..

[18] Wilby RL (1998). Statistical downscaling of general circulation model output: A comparison of methods. Water Resour. Res..

[19] Wood AW, Leung LR, Sridhar V, Lettenmaier DP (2004). Hydrologic Implications of Dynamical and Statistical Approaches to Downscaling Climate Model Outputs. Clim. Change.

[20] Jiang Y (2018). Inter-comparison of multiple statistically downscaled climate datasets for the Pacific Northwest, USA. Sci. Data.

[21] Dixon KW (2016). Evaluating the stationarity assumption in statistically downscaled climate projections: is past performance an indicator of future results?. Clim. Change.

[22] Walton DB, Hall A, Berg N, Schwartz M, Sun F (2017). Incorporating Snow Albedo Feedback into Downscaled Temperature and Snow Cover Projections for California’s Sierra Nevada. J. Clim..

[23] Dieng, D. *et al*. Multivariate Bias‐Correction of High‐Resolution Regional Climate Change Simulations for West Africa: Performance and Climate Change Implications. *J. Geophys. Res. Atmospheres* **127**, (2022).

[24] Rummukainen M (2010). State‐of‐the‐art with regional climate models. WIREs Clim. Change.

[25] Ashfaq M (2016). High-resolution ensemble projections of near-term regional climate over the continental United States: CLIMATE PROJECTIONS OVER THE U.S. J. Geophys. Res. Atmospheres.

[26] Meyer JDD, Wang S‐YS, Gillies RR, Yoon J (2021). Evaluating NA‐CORDEX historical performance and future change of western U.S. precipitation patterns and modes of variability. Int. J. Climatol..

[27] Rastogi, D., Kao, S. & Ashfaq, M. How May the Choice of Downscaling Techniques and Meteorological Reference Observations Affect Future Hydroclimate Projections? *Earths Future* **10**, (2022).

[28] Srivastava AK, Grotjahn R, Ullrich PA, Zarzycki C (2022). Evaluation of precipitation indices in suites of dynamically and statistically downscaled regional climate models over Florida. Clim. Dyn..

[29] McGinnis S, Mearns L (2021). Building a climate service for North America based on the NA-CORDEX data archive. Clim. Serv..

[30] Malek K (2022). Bias Correction of Hydrologic Projections Strongly Impacts Inferred Climate Vulnerabilities in Institutionally Complex Water Systems. J. Water Resour. Plan. Manag..

[31] Xue, Z. & Ullrich, P. A Retrospective and Prospective Examination of the 1960s U.S. Northeast Drought. *Earths Future* **9**, (2021).

[32] Mahoney K (2018). An Examination of an Inland-Penetrating Atmospheric River Flood Event under Potential Future Thermodynamic Conditions. J. Clim..

[33] Patricola CM, Wehner MF (2018). Anthropogenic influences on major tropical cyclone events. Nature.

[34] Ullrich PA (2018). California’s Drought of the Future: A Midcentury Recreation of the Exceptional Conditions of 2012–2017. Earths Future.

[35] Liu C (2017). Continental-scale convection-permitting modeling of the current and future climate of North America. Clim. Dyn..

[36] Vahmani P, Jones AD, Patricola CM (2019). Interacting implications of climate change, population dynamics, and urban heat mitigation for future exposure to heat extremes. Environ. Res. Lett..

[37] Bercos-Hickey E (2022). Anthropogenic Contributions to the 2021 Pacific Northwest Heatwave. Geophys. Res. Lett..

[38] Schär C, Frei C, Lüthi D, Davies HC (1996). Surrogate climate-change scenarios for regional climate models. Geophys. Res. Lett..

[39] Saha S (2010). The NCEP Climate Forecast System Reanalysis. Bull. Am. Meteorol. Soc..

[40] Hersbach H (2020). The ERA5 global reanalysis. Q. J. R. Meteorol. Soc..

[41] Navarro-Racines C, Tarapues J, Thornton P, Jarvis A, Ramirez-Villegas J (2020). High-resolution and bias-corrected CMIP5 projections for climate change impact assessments. Sci. Data.

[42] Bloomfield HC, Brayshaw DJ, Deakin M, Greenwood D (2022). Hourly historical and near-future weather and climate variables for energy system modelling. Earth Syst. Sci. Data.

[43] Reed KA, Stansfield AM, Wehner MF, Zarzycki CM (2020). Forecasted attribution of the human influence on Hurricane Florence. Sci. Adv..

[44] Shepherd TG (2018). Storylines: an alternative approach to representing uncertainty in physical aspects of climate change. Clim. Change.

[45] Wehner MF, Reed KA (2022). Operational extreme weather event attribution can quantify climate change loss and damages. PLOS Clim..

[46] Trenberth KE, Fasullo JT, Shepherd TG (2015). Attribution of climate extreme events. Nat. Clim. Change.

[47] Hall A (2014). Projecting regional change. Science.

[48] Rastogi, D., Lehner, F. & Ashfaq, M. Revisiting Recent U.S. Heat Waves in a Warmer and More Humid Climate. *Geophys. Res. Lett*. **47**, (2020).

[49] Gutmann ED (2018). Changes in Hurricanes from a 13-Yr Convection-Permitting Pseudo–Global Warming Simulation. J. Clim..

[50] Rasmussen KL, Prein AF, Rasmussen RM, Ikeda K, Liu C (2020). Changes in the convective population and thermodynamic environments in convection-permitting regional climate simulations over the United States. Clim. Dyn..

[51] Reed, P. M. *et al*. Multisector Dynamics: Advancing the Science of Complex Adaptive Human‐Earth Systems. *Earths Future* **10**, (2022).

[52] AghaKouchak A (2020). Climate Extremes and Compound Hazards in a Warming World. Annu. Rev. Earth Planet. Sci..

[53] Dong, L. *et al*. Meteorological Environments Associated With California Wildfires and Their Potential Roles in Wildfire Changes During 1984–2017. *J. Geophys. Res. Atmospheres* **126** (2021).

[54] Deser C, Phillips AS, Alexander MA, Smoliak BV (2014). Projecting North American Climate over the Next 50 Years: Uncertainty due to Internal Variability. J. Clim..

[55] Maher N, Milinski S, Ludwig R (2021). Large ensemble climate model simulations: introduction, overview, and future prospects for utilising multiple types of large ensemble. Earth Syst. Dyn..

[56] Rahimi, S. *et al*. Evaluation of a Reanalysis‐Driven Configuration of WRF4 Over the Western United States From 1980 to 2020. *J. Geophys. Res. Atmospheres* **127**, (2022).

[57] Skamarock, W. C. *et al*. A Description of the Advanced Research WRF Version 3.

[58] Ashfaq, M., Rastogi, D., Kitson, J., Abid, M. A. & Kao, S. Evaluation of CMIP6 GCMs Over the CONUS for Downscaling Studies. *J. Geophys. Res. Atmospheres* **127**, (2022).

[59] Zelinka MD (2020). Causes of Higher Climate Sensitivity in CMIP6 Models. Geophys. Res. Lett..

[60] Hausfather Z, Marvel K, Schmidt GA, Nielsen-Gammon JW, Zelinka M (2022). Climate simulations: recognize the ‘hot model’ problem. Nature.

[61] Forster, *et al*. The Earth’s Energy Budget, Climate Feedbacks, and Climate Sensitivity. In Climate Change 2021: The Physical Science Basis. *Contribution of Working Group I to the Sixth Assessment Report of the Intergovernmental Panel on Climate Change* [Masson-Delmotte, V. *et al*. (eds.)]. Cambridge University Press, Cambridge, United Kingdom and New York, NY, USA, 923–1054, (2021).

[62] Ek, M. B. *et al*. Implementation of Noah land surface model advances in the National Centers for Environmental Prediction operational mesoscale Eta model. *J. Geophys. Res. Atmospheres* **108**, (2003).

[63] Fry JE (2011). Completion of the 2006 National Land Cover Database for the conterminous United States. Photogramm. Eng. Remote Sens..

[64] Wickham JD (2013). Accuracy assessment of NLCD 2006 land cover and impervious surface. Remote Sens. Environ..

[65] Yang J (2015). Enhancing Hydrologic Modelling in the Coupled Weather Research and Forecasting–Urban Modelling System. Bound.-Layer Meteorol..

[66] Kusaka H, Kondo H, Kikegawa Y, Kimura F (2001). A Simple Single-Layer Urban Canopy Model For Atmospheric Models: Comparison With Multi-Layer And Slab Models. Bound.-Layer Meteorol..

[67] Chen F (2011). The integrated WRF/urban modelling system: development, evaluation, and applications to urban environmental problems. Int. J. Climatol..

[68] Qian Y (2022). Urbanization Impact on Regional Climate and Extreme Weather: Current Understanding, Uncertainties, and Future Research Directions. Adv. Atmospheric Sci..

[69] Kalnay E, Cai M (2003). Impact of urbanization and land-use change on climate. Nature.

[70] Wang J, Kotamarthi VR (2013). Assessment of Dynamical Downscaling in Near-Surface Fields with Different Spectral Nudging Approaches Using the Nested Regional Climate Model (NRCM). J. Appl. Meteorol. Climatol..

[71] Zobel Z, Wang J, Wuebbles DJ, Kotamarthi VR (2017). High‐Resolution Dynamical Downscaling Ensemble Projections of Future Extreme Temperature Distributions for the United States. Earths Future.

[72] Spero TL, Otte MJ, Bowden JH, Nolte CG (2014). Improving the representation of clouds, radiation, and precipitation using spectral nudging in the Weather Research and Forecasting model: Spectral Nudging of Moisture in WRF. J. Geophys. Res. Atmospheres.

[73] Spero TL, Nolte CG, Mallard MS, Bowden JH (2018). A Maieutic Exploration of Nudging Strategies for Regional Climate Applications Using the WRF Model. J. Appl. Meteorol. Climatol..

[74] Meinshausen M (2017). Historical greenhouse gas concentrations for climate modelling (CMIP6). Geosci. Model Dev..

[75] Jones AD (2023). MSD-LIVE.

[76] Daly C (2008). Physiographically sensitive mapping of climatological temperature and precipitation across the conterminous United States. Int. J. Climatol..

[77] Reed KA, Wehner MF, Zarzycki CM (2022). Attribution of 2020 hurricane season extreme rainfall to human-induced climate change. Nat. Commun..

[78] Ullrich PA, Zarzycki CM (2017). TempestExtremes: a framework for scale-insensitive pointwise feature tracking on unstructured grids. Geosci. Model Dev..

[79] Ullrich PA (2021). TempestExtremes v2.1: a community framework for feature detection, tracking, and analysis in large datasets. Geosci. Model Dev..

[80] Stansfield AM, Reed KA, Zarzycki CM, Ullrich PA, Chavas DR (2020). Assessing Tropical Cyclones’ Contribution to Precipitation over the Eastern United States and Sensitivity to the Variable-Resolution Domain Extent. J. Hydrometeorol..

[81] Knapp KR, Kruk MC, Levinson DH, Diamond HJ, Neumann CJ (2010). The International Best Track Archive for Climate Stewardship (IBTrACS): Unifying Tropical Cyclone Data. Bull. Am. Meteorol. Soc..

[82] Shaevitz DA (2014). Characteristics of tropical cyclones in high-resolution models in the present climate. J. Adv. Model. Earth Syst..

[83] Jones E, Wing AA, Parfitt R (2021). A Global Perspective of Tropical Cyclone Precipitation in Reanalyses. J. Clim..

[84] Xie P (2007). A Gauge-Based Analysis of Daily Precipitation over East Asia. J. Hydrometeorol..

[85] Bloch-Johnson J, Rugenstein M, Gregory J, Cael BB, Andrews T (2022). Climate impact assessments should not discount ‘hot’ models. Nature.

[86] Hausfather Z, Peters GP (2020). Emissions – the ‘business as usual’ story is misleading. Nature.

[87] Lawrence J, Haasnoot M, Lempert R (2020). Climate change: making decisions in the face of deep uncertainty. Nature.

[88] Thompson G, Field PR, Rasmussen RM, Hall WD (2008). Explicit Forecasts of Winter Precipitation Using an Improved Bulk Microphysics Scheme. Part II: Implementation of a New Snow Parameterization. Mon. Weather Rev..

[89] Morrison H, Milbrandt JA (2015). Parameterization of Cloud Microphysics Based on the Prediction of Bulk Ice Particle Properties. Part I: Scheme Description and Idealized Tests. J. Atmospheric Sci..

[90] Thompson G, Eidhammer T (2014). A Study of Aerosol Impacts on Clouds and Precipitation Development in a Large Winter Cyclone. J. Atmospheric Sci..

[91] Tiedtke M (1989). A Comprehensive Mass Flux Scheme for Cumulus Parameterization in Large-Scale Models. Mon. Weather Rev..

[92] Iacono MJ (2008). Radiative forcing by long-lived greenhouse gases: Calculations with the AER radiative transfer models. J. Geophys. Res..

[93] Janjić ZI (1994). The Step-Mountain Eta Coordinate Model: Further Developments of the Convection, Viscous Sublayer, and Turbulence Closure Schemes. Mon. Weather Rev..

[94] Mesinger F (2010). Several PBL parameterization lessons arrived at running an NWP model. IOP Conf. Ser. Earth Environ. Sci..

[95] Hong S-Y, Noh Y, Dudhia J (2006). A New Vertical Diffusion Package with an Explicit Treatment of Entrainment Processes. Mon. Weather Rev..

[96] Monin AS, Obukhov AM (1954). Basic laws of turbulent mixing in the surface layer of the atmosphere. Tr. Akad. Nauk SSSR Geophiz. Inst..

[97] Jiménez PA (2012). A Revised Scheme for the WRF Surface Layer Formulation. Mon. Weather Rev..

[98] Niu, G.-Y. *et al*. The community Noah land surface model with multiparameterization options (Noah-MP): 1. Model description and evaluation with local-scale measurements. *J. Geophys. Res. Atmospheres* **116**, (2011).

